# Novel Terpenoids with Potent Cytotoxic Activities from *Resina Commiphora*

**DOI:** 10.3390/molecules23123239

**Published:** 2018-12-07

**Authors:** Bin-Yuan Hu, Da-Peng Qin, Shao-Xiang Wang, Jing-Jing Qi, Yong-Xian Cheng

**Affiliations:** 1 School of Chemical Science and Technology, Yunnan University, Kunming 650091, China; HuBinyuan2018@163.com; 2 Guangdong Key Laboratory for Genome Stability & Disease Prevention, School of Pharmaceutical Sciences, Shenzhen University Health Science Center, Shenzhen 518060, China; tqindp@szu.edu.cn (D.-P.Q.); wsx@szu.edu.cn (S.-X.W.); qijing023@163.com (J.-J.Q.)

**Keywords:** *Resina Commiphora*, terpenoids, X-ray, quantum chemical computation, cytotoxic activities

## Abstract

A novel sesquiterpene dimer, spirocommiphorfuran A (**1**); two new cadinane sesquiterpenoids, commiphorenes A (**2**) and B (**3**); along with three known terpenoids (**4**–**6**), were isolated from *Resina* *Commiphora*. The structures of these new compounds were characterized by NMR, HRESIMS, quantum chemical computation, and X-ray diffraction analysis. Compound **1** features a 7-oxabicyclo[2.2.1]heptane-2-ene core, representing the first example of germacrane-type sesquiterpene dimer fused via a spiro ring system. Compound **2** is a novel sesquiterpene with a completely new carbon skeleton, which is characteristic of an additional carbon attaching to the cadinane backbone via a carbon–carbon bond. Additionally, compounds **2** and **4** exert acceptable cytotoxicity toward normal cells and high selectivity in cancer cells, especially in HepG2 cells.

## 1. Introduction

The *Commiphora* genus in the family Burseraceae comprises over 150 species, spreading over the tropical and subtropical regions, especially occurring in Eastern Africa, Arabia, and India [1]. The resins from the bark of *Commiphora* plants, also known as myrrh, have been historically used as a principal folk in Ayurvedic medicine, traditional Chinese medicine, and other indigenous medical systems for the treatment of arthritis, hyperlipidemia, pain, wounds, fractures, and diseases caused by blood stagnation [2,3]. Chemical investigations on myrrh revealed the constituents to be terpenoids, flavonoids, lignans, steroids, and carbohydrates [4]; some of these constituents were reported to have biological activities, such as cytotoxic [5], anesthetic [6], anti-inflammatory [7], and antimicrobial effects [8,9]. In our previous research of the bioactive terpenoids from *Resina Commiphora*, novel compounds were isolated [10,11,12,13,14]. In the course of our continuing investigations on structurally diverse and biologically intriguing terpenoids from *Resina Commiphora*, three new terpenoids (**1**–**3**), along with three known analogues (**4**–**6**), were isolated. Interestingly, compound **1** is an unexpected sesquiterpene dimer bearing a rare 7-oxabicyclo[2.2.1]heptane-2-ene core and a spiro ring system [15], and compound **2** is an unusual cadinane sesquiterpene containing an additional carbon. Compounds **2** and **4** inhibit cancer cells growth with low cytotoxicity in normal cells.

## 2. Results and Discussion

### 2.1. Structure Elucidation of the Compounds

**Figure 1:**
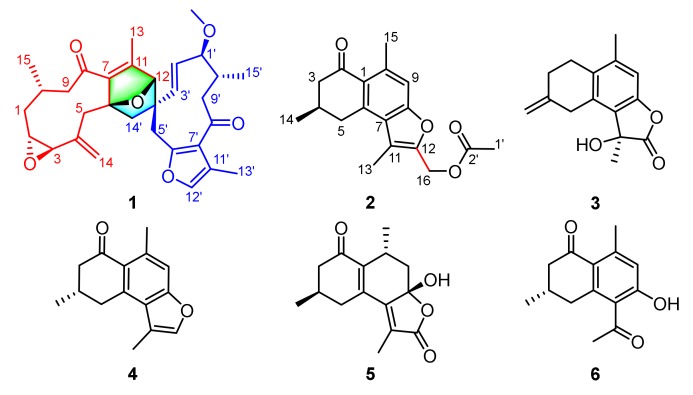
The structures of compounds **1**–**6** from *Resina Commiphora*.

**Figure 2:**
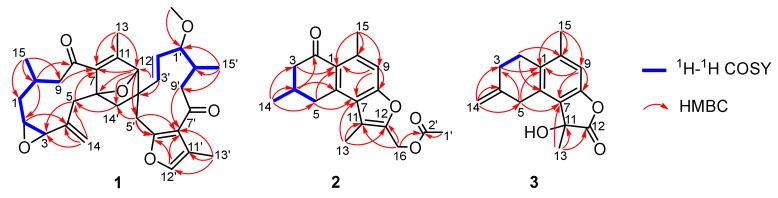
Key ^1^H-^1^H COSY and HMBC correlations for (**1**–**3**).

Spirocommiphorfuran A (**1**), obtained as colorless block crystals, has a molecular formula C_31_H_38_O_6_ (13 degrees of unsaturation) on the basis of its HRESIMS (*m*/*z* 507.2736, calcd. 507.2741 [M + H]^+^), ^13^C-NMR, and DEPT spectra. The ^1^H-NMR data (Table 1) of **1** exhibit four methyl (δ_H_ 2.07 (s), 2.05 (s), 1.13 (s), and 1.08 (d, *J* = 6.2 Hz)), one methoxy (δ_H_ 3.18 (s)), and five olefinic methine protons (δ_H_ 7.06 (s), 5.10 (overlap), 5.08 (overlap), 4.92 (overlap), and 4.88 (overlap)). The ^13^C-NMR and DEPT spectra (Table 1) display 31 carbon resonances, including four methyl, one methoxy, seven methylene (one olefinic), nine methine (three olefinic with one oxygenated, and six aliphatic with four oxygenated), and ten quaternary carbons (two keto-carbonyls, six olefinic, including one oxygenated, and two aliphatic with one oxygenated). In consideration of the aforementioned data and the chemical profile of the genus *commiphora*, we speculated that **1** might be a sesquiterpene dimer. The structural architecture of **1** (Figure 1) was first elucidated by using 2D-NMR. The ^1^H-^1^H COSY spectrum of **1** shows the following correlations: H_2_-9/H-10/H_2_-1/H-2/H-3 and H-10/H_3_-15 (Figure 2). Starting from this spin system, the observed HMBC correlations of H-2/C-4, H-3/C-5, H_2_-5/C-3, C-7, C-14, H-9/C-7, H-10/C-8, H_3_-13/C-7, C-11, C-12, H_2_-14/C-3, C-4, C-5, H_3_-15/C-1, C-9, and C-10 (Figure 2) allowed us to conclude the presence of a germacrane-type sesquiterpene moiety; apart from these characteristic signals, an epoxy group is also observed in the 1D-NMR spectra, whose position is assigned to C-2 (δ_C_ 62.6) and C-3 (δ_C_ 61.1) due to the observation of the HMBC correlations of H-10/C-2 and H_2_-1, H_2_-5, and H_2_-14/C-3 (Figure 2). Thus, part I of **1** was deduced as shown (red lines in Figure 1). Part II (blue lines in Figure 1) was established by analysis of the remaining carbon signals in the ^13^C-NMR spectrum. Further studies suggest that part II is similar to those reported for the related furanogermacren derivatives [16]; the main difference is that the *Δ*^4′(14′)^ double bond is absent in part II. Parts I and II are connected via C-6‒C-14′ and C-12‒C-4′ supported by the HMBC correlations of H-12/C-3′, C-4′, C-5′, C-14′, H_2_-5/C-14′, H_2_-14′/C-5, C-6, C-7, H-3′, and H-5′/C-12. The presence of an oxygen bridge between C-6 and C-12 is supported by the chemical shifts of C-6 (δ_C_ 91.2), C-12 (δ_C_ 88.2), the HMBC correlation of H-12/C-6, the requirement of the molecular formula, and the remaining one degree of unsaturation. Therefore, the plane structure of **1** was deduced.

**Figure 3:**
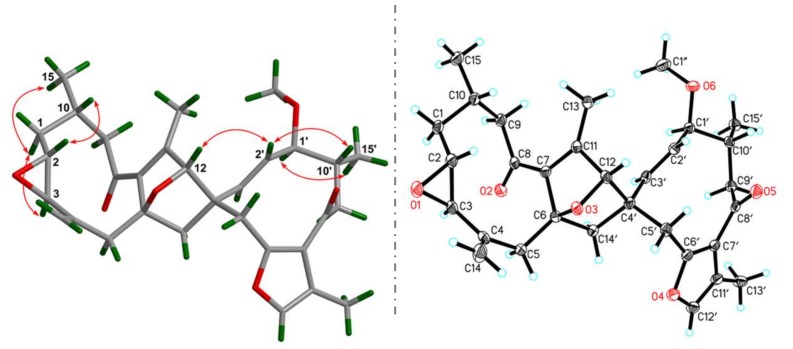
Key ROESY correlations and X-ray crystallographic analysis for **1**.

The ROESY correlations (Figure 3) of H-2/H-10, Ha-1/H-3, and H_3_-15 are observed, indicating the relative configurations at C-2, C-3, and C-10. Meanwhile, H-2′ interacting with H-12 in the ROESY spectrum, in consideration of the rigidity of the bridged ring, allows one to assign the stereochemistry of the bicyclo[2.2.1]heptane ring. Finally, the ROESY correlation of H-1′/H_3_-15′ suggests that these protons are spatially vicinal. Due to the flexibility of two ten-membered rings, it is hard to detect the ROESY restrictions between these two middle rings. Fortunately, X-ray crystallographic analysis of **1** with the excellent Flack parameter [0.00(11)] allows one to clarify the absolute configurations at each chiral center in the architecture (Figure 3). In general, the geometry of *Δ*^2′(3′)^ double bond could be concluded from the coupling constant of H-2′ or H-3′. However, this is impossible in the present structure due to the overlapped signals for these protons. The X-ray data also indicate an *E* configuration for the *Δ*^2′(3′)^ double bond. Collectively, the structure of **1** was deduced as 2*R*,3*R*,6*R*,10*S*,12*S*,1′*S*,4′*S*,10′*R.*

Commiphorene A (**2**), obtained as yellow gums, was found to have the molecular formula C_18_H_20_O_4_ by analysis of its HRESIMS, ^13^C-NMR, and DEPT spectra, indicating nine degrees of unsaturation. The ^1^H-NMR spectrum of **2** exhibits four methyl groups (δ_H_ 2.71 (s), 2.47 (s), 2.10 (s), and 1.18 (d, *J* = 6.2 Hz)), and one olefinic/aromatic proton. The ^13^C-NMR and DEPT spectra (Table S1) reveal 18 carbon signals classified into four methyl, three methylene, two methine (one olefinic), and nine quaternary carbons (one ketone-carbonyl, one ester carbonyl, and seven olefinic, including two oxygenated). The structure of **2** was mainly assembled by interpretation of its 2D-NMR data. The ^1^H-^1^H COSY spectrum discloses interactions of H-3/H-4/H-5 and H-4/H_3_-14. This spin system, in combination with the HMBC correlations of H-3/C-1, C-2 (δ_C_ 195.6), H-4/C-2, C-6 and H-5/C-1, and C-6 reveals the presence of a six-membered ring (C-1−C-2−C-3−C-4−C-5−C-6) with a ketone-carbonyl group at C-2 and a methyl group at C-4, as shown (Figure 1). Further, HMBC correlations of H-5/C-1 (δ_C_ 126.8), C-6 (δ_C_ 140.9) and C-7 (δ_C_ 124.7), H-9/C-1, C-7, C-8 (δ_C_ 156.2), C-10 (δ_C_ 139.5) and C-15 (δ_C_ 24.3), and H_3_-15/C-1, C-9 (δ_C_ 113.1) and C-10 reveal a pentasubstituted benzene ring (C-6−C-7−C-8−C-9−C-10−C-1) with a methyl group at C-10. The associations of H_3_-13/C-7, C-11 (δ_C_ 116.7), C-12 (δ_C_ 147.7) and H_2_-16/C-11, and C-12 indicate the presence of a chain consisting of C-13−C-11−C-12−C-16 connected with the pentasubstituted benzene ring via the carbon bond C-7−C-11. Furthermore, the presence of an acetoxyl group positioned at C-16 is supported by the chemical shift of C-16 (δ_C_ 56.3), as well as the HMBC correlations of H_2_-16/C-2′ and H_3_-1′/C-2′. In consideration of the chemical shifts of C-8 (δ_C_ 156.2) and C-12 (δ_C_ 147.7), the requirement of the molecular formula, and the remaining one degree of unsaturation, the presence of C-8−*O*−C-12 is secured. Taken together, the planar structure of **2** was identified.

**Figure 4:**
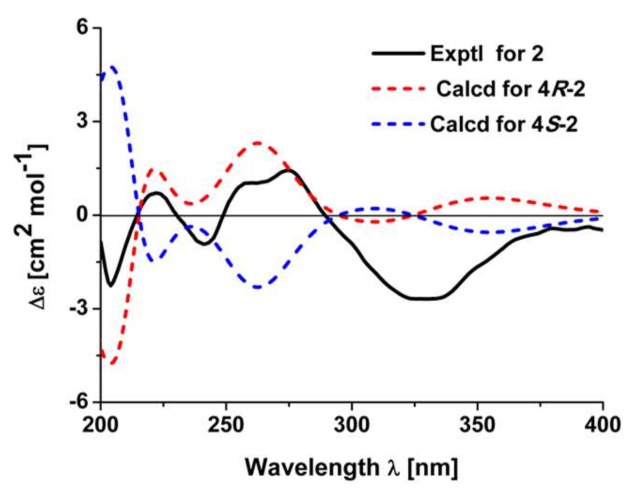
Experimental electronic circular dichroism (ECD) spectrum of **2** and calculated ECD spectra of 4*R*-**2** and 4*S*-**2**.

It was noted that there is only one chiral center in **2**. The absolute configuration of **2** was verified by the quantum chemical electronic circular dichroism (ECD) calculation using the TDDFT method. The experimental ECD spectrum of **2** is similar to the calculated one of (4*R*)-**2** (Figure 4). Thus, the structure of **2** was finally deduced.

Commiphorene B (**3**) was obtained as white gums. Its molecular formula was deduced as C_15_H_16_O_3_ (8 degrees of unsaturation) by its HREIMS (*m*/*z* 244.1103, calcd for C_15_H_16_O_3_ 244.1099), ^13^C-NMR, and DEPT spectra. The ^1^H-NMR spectrum of **3** (Table 2) exhibits two methyl groups (δ_H_ 2.25 (s) and 1.79 (s)), one olefinic methylene (δ_H_ 4.90 (2H, overlap)), and one olefinic methine. The ^13^C-NMR and DEPT spectra of **3** (Table 2) display 15 carbons classified into two methyl, four methylene (one olefinic), one olefinic methine, and eight quaternary carbons (one ester carbonyl and six olefinic, including one oxygenated). Inspection of these NMR data found that the signals of **3** (Table 2) resemble those of (11β)-8-11-dihydroxycadina-6,8,10-trien-12-oic acid *γ*-lactone [17], differing only in that a *Δ*^(4,14)^ double bond is present in **3**, instead of a methyl group at C-4; the drastic downfield shifts of C-4 (δ_C_ 143.7) and C-14 (δ_C_ 109.0) and the HMBC correlations (Figure 2) of H_2_-2/C-4, H_2_-3, and H_2_-5/C-14 secure the conclusion.

The absolute configuration of **3** was determined by optical rotation (OR) calculation. The Merck Molecular Force Field (MMFF) conformational search of (11-*R*)-**3** and (11*S*)-**3** resulted in 2 and 2 conformers in a 17 kJ/mol energy window, respectively. These conformers were further optimized by TDDFT at the B3LYP/6-31G(d) level, as implemented in the Gaussian 09 program package [18]. Subsequently, the stable conformers (Figure 3) obtained for each enantiomer were submitted to theoretical OR calculations at the B3LYP/6 – 311 + G(d,p) level. The resulting OR values of (11-*R*)-**3** and (11*S*)-**3** were energetically weighted according to the respective conformational distribution by the Boltzmann statistics.

The OR values of (11-*R*)-**3** and its enantiomer (11*S*)-**3** were predicted to be +28.8 and −29.6, respectively. The OR value for compound (11*S*)-**3** is closer to the experimental one (−21.6). Therefore, the absolute configuration of **3** was proposed to be 11*S*.

The known compounds were identified as myrrhone [19], commipholinone [20], and myrrhanolide C [21], respectively, by comparing their spectroscopic data with those reported in the literature.

### 2.2. Biological Evaluation

All of the compounds were evaluated for their cytotoxic activities against human cancer cells originating from the liver, gastric, and lung. All cells were exposed to various concentrations (0–160 μM) for 48 h, and cell viability was quantified by CCK-8 assay (Table 3). As a result, only compounds **2**, **4**, and **5**-**FU** (positive control) significantly inhibited the growth of four human cancer cell lines in a dose-dependent manner, and the other compounds were not active (data not shown). Further, we found that the IC_50_ values of **2** against their cancer cells are from 48.67 μM to 152.50 μM, and the most sensitive cell line is HepG2, in which **2** and **4** are more potent than **5**-**FU**. On the other hand, the IC_50_ value could not be determined, even at a concentration of up to 160 μM in normal human umbilical vein endothelial cells, suggesting that **2** and **4** exert acceptable cytotoxicity in normal cells and high selectivity in cancer cells, especially in HepG2 cells.

## 3. Experimental Section

### 3.1. General Procedures

Column chromatography was undertaken on silica gel (200–300 mesh, Qingdao Marine Chemical Inc., Qingdao, China), MCI gel CHP 20P (75–150 μm, Mitsubishi Chemical Industries, Tokyo, Japan), RP-18 (40–60 µm; Daiso Co., Tokyo, Japan), and Sephadex LH-20 (Amersham Pharmacia, Uppsala, Sweden). Optical rotations were measured on a Bellingham + Stanley ADP 440 + digital polarimeter (Bellingham & Stanley, Kent, UK). UV spectra were obtained on a Shimadzu UV-2600 spectrometer (Shimadzu Corporation, Tokyo, Japan). CD spectra were measured on a Chirascan instrument (Agilent Technologies, Santa Clara, CA, USA). Semi-preparative or analytic HPLC was carried out using an Agilent 1200 liquid chromatograph (Agilent Technologies, Santa Clara, CA, USA). The column used was a YMC-Pack ODS-A 250 mm × 9.4 mm, i.d., 5 µm, or a Thermo Hypersil GOLD-C18 250 mm × 21.2 mm, i.d., 5 µm. NMR spectra were recorded at room temperature on a Bruker AV-800 spectrometer (Bruker, Karlsruhe, Germany) with TMS as an internal standard. HRESIMS of compounds **1** and **2** were collected by a Shimazu LC-20AD AB SCIEX triple TOF 5600 + MS spectrometer (Shimadzu Corporation, Tokyo, Japan). HRESIMS data of **3** was collected by AutoSpec Premier P776 spectrometer (Waters Corporation, Milford, MA, USA).

### 3.2. Plant Material

The medicinal materials of *Resina Commiphora* (myrrh) were obtained from the Juhuacun Market of Material Medica, Kunming, Yunnan Province, China, in July 2013. The material was identified by Mr. Bin Qiu at Yunnan Institute of Materia Medica, and a voucher specimen (CHYX-0585-2) was deposited at School of Pharmaceutical Sciences, Shenzhen University, China in November 2017.

### 3.3. Extraction and Isolation

The dried myrrha (50 kg) was ground and soaked with 95% EtOH (180 L, 3 × 48 h) to give a crude extract, which was suspended in warm water, followed by extraction with EtOAc to afford an EtOAc soluble extract (8 kg). This extract was divided into six parts (Fr.A–Fr.F) by using a silica gel column eluted with petroleum ether–acetone (100:0, 100:1, 60:1, 40:1, 20:1, 5:1, 3:1, 1:1, and 0:100). Fr.B (2.4 kg) was further separated via a silica gel column washed with petroleum ether–EtOAc (100:0, 99:1, 60:1, 40:1, 20:1, 5:1, 3:1, and 1:1) and petroleum ether–acetone (5:1, 3:1, and 1:1) to provide six portions (Fr.B.1–Fr.B.6). Fr.B.5 (186.6 g) was separated via MCI gel CHP 20P eluted with aqueous MeOH (55–100%) to provide eight portions (Fr.B.5.1–Fr.B.5.8). Fr.B.5.5 (16.0 g) was subjected to a RP-18 column eluted with aqueous MeOH (50–100%) to yield nine fractions (Fr.B.5.5.1−Fr.B.5.5.9). Fr.B.5.5.7 (4.03 g) was passed through Sephadex LH-20 (MeOH) to yield five fractions (Fr.B.5.5.7.1–Fr.B.5.5.7.5). Fr.B.5.5.7.3 (560 mg) was divided into four fractions (Fr.B.5.5.7.3.1–Fr.B.5.5.7.3.4) by using silica gel chromatography (petroleum ether–acetone, 30:1, 25:1, 20:1, 15:1, 10:1, 5:1, and 1:1). Fr.B.5.5.7.3.3 (12 mg) was further purified by semi-preparative HPLC with aqueous MeOH (82%) to afford compound **1** (3.2 mg, t_R_ = 15.4 min; flow rate: 3 mL/min). Fr.B.5.7 (17 g) was subjected to a RP-18 column washed with aqueous MeOH (40–100%) to provide 14 portions (Fr.B.5.7.1–Fr.B.5.7.14). Fr.5.7.3 (252.7 mg) was subjected to preparative TLC (CHCl_3_–EtOAc–HCOOH (4:1 drops) to give Fr.5.7.3.1–Fr.5.7.3.9). Compound **6** (5.5 mg, t_R_ = 9.7 min; flow rate: 3 mL/min) was from Fr.5.7.3.3 (43.9 mg) by semi-preparative HPLC (aqueous MeOH, 70%). Fr. B.5.7.5 (510.7 mg) was submitted to Sephadex LH-20 (MeOH) to yield three fractions (Fr.B.5.7.5.1−Fr.B.5.7.5.3). Fr.B.5.7.5.3 (20.0 mg) was purified by semi-preparative HPLC (aqueous MeCN, 55%) to give two portions. Compound **3** (0.8 mg) was obtained from Fr.B.5.7.5.3.2 (2.1 mg, t_R_ = 12.9 min, flow rate: 3 mL/min) by HPLC separation (aqueous MeOH, 78%). Fr.B.5.7.7 (452.5 mg) was passed through Sephadex LH-20 (MeOH) to yield two fractions (Fr.B.5.7.7.1–Fr.B.5.7.7.2). Further purification of Fr.B.5.7.7.2 (146.2 mg) by semi-preparative HPLC eluted with aqueous MeCN (67%) afforded compound **2** (1.2 mg, t_R_ = 17.3 min; flow rate: 3 mL/min). Fr.5.7.8 (1.2g) was submitted to Sephadex LH-20 (MeOH) to yield five fractions (Fr.B.5.7.8.1−Fr.B.5.7.8.5). Fr.5.7.8.4 (110 mg) was purified by semi-preparative HPLC (aqueous MeCN, 68%) to yield Compound **4** (5.1 mg, t_R_ = 20.8 min; flow rate: 3 mL/min). Fr.B.5.8 (25.8 g) was separated via RP-18 eluted with aqueous MeOH (50–100%) to provide ten portions (Fr.B.5.8.1–Fr.B.5.8.10). Fr.5.8.1 (220 mg) was purified by semi-preparative HPLC (aqueous MeCN, 40%) to give four portions. Compound **5** (9.9 mg) was obtained from Fr.B.5.8.1.4 (15.2 mg, t_R_ = 16.2 min, flow rate: 3 mL/min) by HPLC separation (aqueous MeCN, 37%).

### 3.4. Compound Characterization Data

Compound **1**: Colorless block crystals (CH_3_OH), ${\left[\alpha \right]}_{D}^{25}$ + 9.6 (*c* 0.18, CH_3_OH); UV (CH_3_OH) λ_max_ (log *ε*) 252 (5.32) nm; HRESIMS: *m*/*z* 507.2736 [M + H]^+^ (calcd. for C_31_H_39_O_6_, 507.2741). ^1^H- and ^13^C-NMR data, see Table 1.

Compound **2**: Yellow gums, ${\left[\alpha \right]}_{D}^{25}$ + 36.1 (*c* 0.36, CH_3_OH); CD (CH_3_OH), *∆ε*_203_ −2.41, *∆ε*_222_ +0.74, *∆ε*_240_ −0.98, *∆ε*_259_ +1.10, *∆ε*_276_ +1.46, *∆ε*_333_ –2.68, *∆ε*_381_ −0.40; UV (CH_3_OH) λ_max_ (log *ε*) 276 (5.86) nm; HRESIMS: *m*/*z* 301.1428 [M + H]^+^ (calcd. for C_18_H_21_O_4_, 301.1434). ^1^H- and ^13^C-NMR data, see Table 2.

Compound **3**: White gums, ${\left[\alpha \right]}_{D}^{25}$ − 21.6 (*c* 0.07, CH_3_OH); UV (CH_3_OH) λ_max_ (log *ε*) 290 (3.23), 253 (3.48), 206 (4.18) nm; HREIMS: *m*/*z* 244.1103 [M]^+^ (calcd. for C_15_H_16_O_3_, 244.1099). ^1^H- and ^13^C-NMR data, see Table 2.

### 3.5. X-ray Crystallographic Analysis of ***1***

Crystal data for compound **1**: Data were collected using a Sapphire CCD with a graphite monochromated Cu K*α* radiation, λ = 1.54184 Å at 100 K. Crystal data: C_31_H_38_O_6_, *M* = 506.61, space group *P*1 21 1; unit cell dimensions were determined to be a = 10.4149(2) Å, b = 9.5899(2) Å, c = 14.2271(2) Å, α = 90.00°, β = 109.362(2)°, γ = 90.00°, V = 1340.61(5) Å3, Z = 2, Dx = 1.255 g/cm^3^, F (000) = 544.0, and μ (Cu K*α*) = 0.692 mm^−1^, and 8122 reflections were collected until θmax = 73.448°, in which independent unique 4692 reflections were observed (F2 > 4σ (F2)). The structure was solved by direct methods using the SHELXS-97 program and refined by the program SHELXL-97 and full-matrix least squares calculations. In the structure refinements, nonhydrogen atoms were placed on the geometrically ideal positions by the “ride on” method. Hydrogen atoms bonded to oxygen were located by the structure factors with isotropic temperature factors. The final refinement gave *R* = 0.0402(4692), *Rw* = 0.1061(4831), *S* = 1.045, and Flack = 0.00(11). Crystallographic data for structure **1** has been deposited at the Cambridge Crystallographic Data Centre (CCDC 1878959), www.ccdc.cam.ac.uk/data_request/cif.

### 3.6. Quantum Chemical Computations of ***2*** and ***3***

The theoretical calculations of compounds **2** and **3** were performed using Gaussian 09 [18]. Conformation search using molecular mechanics calculations was performed in Discovery Studio 3.5 Client with MMFF force field with 17 kJ/mol (approximately 4 kcal/mol) upper energy limit. The optimized conformation geometries and thermodynamic parameters of all selected conformations were provided. The predominant conformers were optimized at B3LYP/6-31G(d) level. The optimized conformers of **2** were used for the ECD calculation, and the optimized conformers of **3** were used for OR calculation at the B3LYP/6-311+G(d,p) level. The solvent effects were taken into account by the polarizable-conductor calculation model (PCM, methanol as the solvent). Percentages for each conformation are shown in Figures S1–S4.

### 3.7. Cell Viability

Cell lines were obtained from the Cell Bank of China Science Academy (Shanghai, China), maintained in Dulbecco’s modified Eagle’s medium (DMEM) supplemented with 10% fetal bovine serum and 100 U/mL penicillin-streptomycin and incubated at 37 °C in an atmosphere of 5% CO_2_. Cell viability was evaluated using the CCK8 assay kit (Dojindo Laboratories, Tokyo, Japan) according to the manufacturer’s instructions. Exponentially growing cells were seeded at 3–8 × 10^3^ cells per well in 96-well culture plates for 24 h. Cells were exposed to increasing concentrations (0–160 μM) of compounds **1**‒**6**, or **5**-**FU** for 48 h. The equal volume of DMSO was used as the solvent control. CCK8 solution (10 μL) was added to each well and incubated for another 1–4 h. Light absorbance of the solution was measured at 450 nm (Epoch 2; BioTek Instruments, Inc., Winooski, VT, USA). The IC_50_ values were calculated using the PrismPad program (version 5.0, GraphPad Software, San Diego, CA, USA).

## 4. Conclusions

*Resina Commiphora* is commonly used in the traditional Chinese medicine system, in which terpenoids are the main chemical constituents. Inspired by our previous results of structurally novel and biologically intriguing compounds from the material, we conducted an in-depth investigation that resulted in an unusual sesquiterpene dimer with a rare 7-oxabicyclo[2.2.1]heptane-2-ene core (**1**); a sesquiterpene with an additional carbon in the backbone (**2**); and a new cadinane-type sesquiterpene (**3**), adding the structural diversity of sesquiterpenes in nature. Due to the attractive construction features of the isolated compounds, their cytotoxic activities were tested. As a result, compounds **2** and **4** exert low cytotoxicity against normal cells and high selectivity in cancer cells, especially in HepG2 cells, which will be helpful for gaining deep insight into the biological role of such terpenoids in human diseases.

## Figures and Tables

**Table 1:** ^1^H- (800 MHz) and ^13^C-NMR (200 MHz) data of **1** in CDCl_3_ (δ in ppm, *J* in Hz).

1					
Position	δ_H_	δ_C_	Position	δ_H_	δ_C_
1	Ha: 2.16 (overlap) *^a^*	40.1 CH_2_	1′	2.84 (m)	88.8, CH
	Hb: 1.01 (m)				
2	2.28 (m)	62.6 CH	2′	4.88 (overlap)	111.7, CH
3	2.80 (s)	61.1, CH	3′	5.08 (overlap)	134.9, CH
4		141.5, C	4′		53.5, C
5	Ha: 3.49 (overlap)	37.7, CH_2_	5′	Ha: 3.20 (overlap)	38.0, CH_2_
	Hb: 2.66 (d, 14.3)			Hb: 3.00 (d, 13.9)	
6		91.2, C	6′		153.9, C
7		151.6, C	7′		128.8, C
8		200.7, C	8′		196.5, C
9	Ha: 2.75 (m)	53.9, CH_2_	9′	Ha: 2.52 (m)	49.1, CH_2_
	Hb: 2.17 (overlap) *^a^*			Hb: 2.42 (m)	
10	2.15 (overlap)	29.3, CH	10′	2.44 (m)	37.2, CH
11		143.8, C	11′		117.6, C
12	4.47 (s)	88.2, CH	12′	7.06 (s)	138.6, CH
13	2.07 (s)	16.7, CH_3_	13′	2.05 (s)	9.5, CH_3_
14	Ha: 5.10 (overlap)	111.7, CH_2_	14′	Ha: 1.97 (overlap)	47.7, CH_2_
	Hb: 4.92 (overlap)			Hb: 1.52 (overlap)	
15	1.08 (d, 6.2)	23.8, CH_3_	15′	1.13 (s)	18.2, CH_3_
			1′-OMe	3.18 (s)	57.0, CH_3_

*^a^* Signals might be interchangeable.

**Table 2:** ^1^H- (800 MHz) and ^13^C-NMR (200 MHz) data of **2** and **3** in CDCl_3_ (δ in ppm, *J* in Hz).

Position	2		3	
	δ_H_	δ_C_	δ_H_	δ_C_
1		126.8, C		132.2, C
2		199.7, C	Ha: 2.76 (m)	28.4, CH_2_
			Hb: 2.71 (m)	
3	Ha: 2.73 (m)	48.7, CH_2_	2.51 (m)	31.6, CH_2_
	Hb: 2.36 (m)			
4	2.32 (overlap)	29.8, CH		143.7, C
5	Ha: 3.54 (m)	35.7, CH_2_	Ha: 3.80 (d, 18.3)	33.4, CH_2_
	Hb: 2.87 (m)		Hb: 3.59 (d, 18.3)	
6		140.9, C		135.1, C
7		124.7, C		123.0, C
8		156.2, C		150.9, C
9	7.15 (s)	113.1, CH	6.79 (s)	110.4, CH
10		139.5, C		139.4, C
11		116.7, C		73.9, C
12		147.7, C		178.4, C
13	2.47 (s)	11.4, CH_3_	1.79 (s)	24.2, CH_3_
14	1.18 (d, 6.2)	21.5, CH_3_	4.90 (overlap)	109.0, CH_2_
15	2.71 (s)	24.3, CH_3_	2.25 (s)	20.6, CH_3_
16	Ha: 5.18 (d, 13.6)	56.3, CH_2_		
	Hb: 5.17 (d, 13.6)			
1′	2.10 (s)	20.8, CH_3_		
2′		170, C		

**Table 3:** IC_50_ values for compounds **2**, **4,** and **5**-**FU** in different cell lines.

Compound	Cell Lines (IC_50_, µM)			
	HepG2	A549	BGC-823	HUVECs
2	48.67 ± 13.38 *	115.40 ± 20.17	152.50 ± 15.63	>160
4	40.34 ± 5.08 *	>160	>160	>160
5-FU (positive control)	76.92 ± 9.08	69.79 ± 5.32	98.19 ± 13.51	107.40 ± 15.08

HepG2: Human liver cancer. A549: Human lung cancer. BGC-823: Human gastric cancer. HUVECs: Normal human umbilical vein endothelial cells [22]. * *P* < 0.05 (vs **5**-**FU** group).

## Supplementary Materials

The following are available online. Figures S1–S4: optimized geometries of predominant conformers for compounds **2** and **3**, Figures S5–S10: NMR spectra of **1**, Figure S11: HREIMS of **1**, Figures S12–S16: NMR spectra of **2**, Figure S17: HRESIMS of **2**, Figures S18–S22: NMR spectra of **3**, Figure S23: HRESIMS of **3**. Table S1–S2: the Cartesian coordinates of the lowest energy conformers for **2** and **3**.
