# Supplementary material for: Novel Terpenoids with Potent Cytotoxic Activities from Resina Commiphora
Source: Molecules. 2018 Dec 7;23(12):3239. doi: 10.3390/molecules23123239 (PMC6321302; doi:10.3390/molecules23123239)
Supplement: Supplementary file 1 [file molecules-23-03239-s001.pdf]

Novel Terpenoids with Potent Cytotoxic Activities from *Resina Commiphora*

Bin-Yuan Hu [1,†], Da-Peng Qin [2,†], Shao-Xiang Wang [2], Jing-Jing Qi [2] and Yong-Xian Cheng [2,*]

[1] School of Chemical Science and Technology, Yunnan University, Kunming 650091, China

[2] School of Pharmacy, Health Science Center, Shenzhen University, Shenzhen 518060, China

[†]These authors contributed equally to this work.

Corresponding author: Tel/Fax: +86-0755-26902073

E-Mail: yxcheng@szu.edu.cn

1. Computational data of **2** and **3**

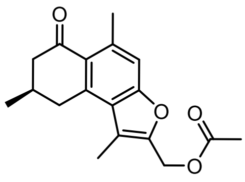

2a

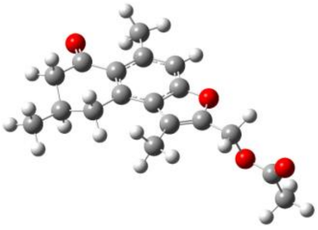

2a-1 (41.51%)

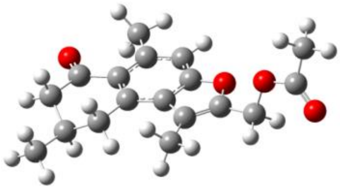

2a-2 (27.78%)

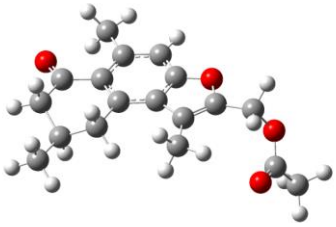

2a-3 (30.71%)

Figure S1. Optimized geometries of predominant conformers for compound **2a**.

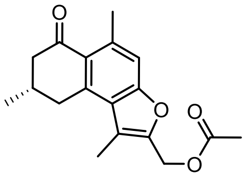

2b

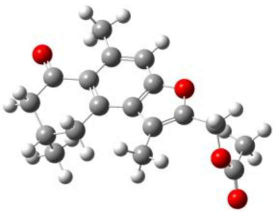

2b-1 (38.72%)

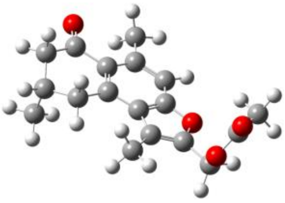

2b-2 (29.55%)

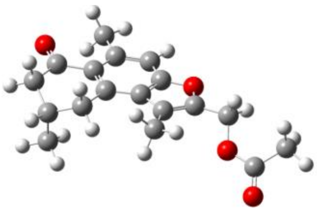

2b-3 (31.73%)

Figure S2. Optimized geometries of predominant conformers for compound **2b**.

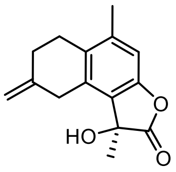

3a

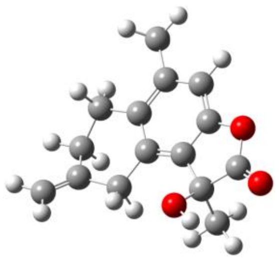

3a-1 (72.65%)

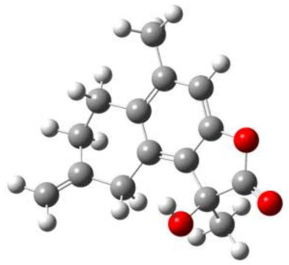

3a-2 (27.35%)

Figure S3. Optimized geometries of predominant conformers for compound **3a**.

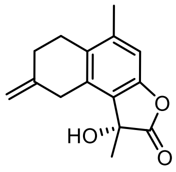

3b

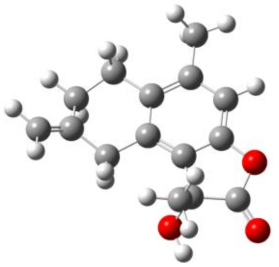

3b-1 (65.86%)

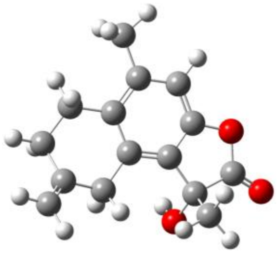

3b-2 (34.14%)

Figure S4. Optimized geometries of predominant conformers for compound **3b**.

Table S1. The Cartesian coordinates of the lowest energy conformers for **2a** and **2b**

**2a-1**	X axis(Å)	Y axis(Å)	Z axis(Å)	**2a-2**	X axis(Å)	Y axis(Å)	Z axis(Å)
O	1.61853	1.91048	0.87313	O	1.50492	2.09273	-0.3394
O	-4.70504	1.06977	0.87962	O	-4.75625	0.87873	-0.20358
C	-2.334	0.82369	0.61573	C	-2.36753	0.72395	-0.35338
C	-1.29019	-0.12141	0.36275	C	-1.26304	-0.185	-0.32477
C	0.05688	0.31934	0.49392	C	0.05135	0.36078	-0.29843
C	0.28918	1.65206	0.80164	C	0.19584	1.73929	-0.35896
C	-0.70264	2.60073	1.01055	C	-0.85554	2.64306	-0.43036
C	-2.03925	2.17736	0.92072	C	-2.16174	2.1258	-0.42268
C	-3.7639	0.34346	0.56923	C	-3.76497	0.15783	-0.289
C	-4.04155	-1.09482	0.19802	C	-3.94659	-1.342	-0.25735

C	-2.96424	-1.66725	-0.70625	C	-2.81003	-2.0675	-0.9558
C	-1.6177	-1.55008	-0.00026	C	-1.49538	-1.67661	-0.28935
C	2.2719	0.74526	0.62382	C	2.23224	0.94832	-0.24946
C	-3.09883	3.22211	1.16822	C	-3.2875	3.128	-0.48365
C	-3.26862	-3.12103	-1.06141	C	-3.01804	-3.5796	-0.90542
C	1.364	-0.27812	0.39442	C	1.39356	-0.15584	-0.21248
C	1.70789	-1.69392	0.10559	C	1.82704	-1.57175	-0.09631
C	3.7561	0.8096	0.66005	C	3.70801	1.11564	-0.20852
O	4.20573	0.52832	-0.66835	O	4.10978	0.82294	1.13232
C	5.55834	0.51637	-0.78794	C	5.45212	0.90077	1.32151
C	5.94639	0.24364	-2.20865	C	5.78746	0.61099	2.75222
O	6.35233	0.69414	0.12447	O	6.2759	1.16138	0.45656
H	-0.43534	3.62538	1.24593	H	-0.65581	3.7083	-0.47657
H	-5.00604	-1.127	-0.323	H	-4.89084	-1.57672	-0.76335
H	-4.13734	-1.67101	1.12591	H	-4.03751	-1.64954	0.79113
H	-2.9324	-1.08554	-1.63804	H	-2.78293	-1.75953	-2.01035
H	-0.85118	-1.94525	-0.67396	H	-0.68647	-2.18944	-0.81864
H	-1.60102	-2.15424	0.91547	H	-1.47227	-2.00744	0.75655
H	-2.65713	4.21495	1.31139	H	-2.90892	4.14903	-0.60815
H	-3.77048	3.30202	0.30777	H	-3.93467	2.92962	-1.34371
H	-3.66193	2.99029	2.07746	H	-3.86512	3.11396	0.4457
H	-2.49809	-3.52788	-1.72471	H	-2.20474	-4.10054	-1.42155
H	-4.23116	-3.20092	-1.5775	H	-3.95766	-3.85841	-1.39388
H	-3.31125	-3.75065	-0.16609	H	-3.052	-3.94416	0.12695
H	1.12109	-2.37173	0.73311	H	1.26914	-2.08453	0.69326
H	1.51368	-1.92649	-0.94599	H	1.66761	-2.0981	-1.04244
H	2.76445	-1.9008	0.30372	H	2.8901	-1.6497	0.15283
H	4.09369	1.80696	0.9645	H	4.18036	0.41693	-0.90863
H	4.14182	0.05912	1.35959	H	3.98832	2.14319	-0.46734
H	5.54225	1.023	-2.85956	H	5.47667	-0.40529	3.007
H	7.03679	0.25004	-2.29416	H	6.86892	0.69174	2.89452
H	5.57828	-0.73997	-2.51088	H	5.29619	1.33873	3.40297
**2a-3**	X axis(Å)	Y axis(Å)	Z axis(Å)	**2b-1**	X axis(Å)	Y axis(Å)	Z axis(Å)
O	1.97307	1.63282	1.01072	O	0.516293	2.362199	0.870211
O	-4.36042	1.8638	0.28944	O	-4.01079	-2.13178	0.868544
C	-2.05821	1.19592	0.36091	C	-1.95586	-0.91254	0.67769
C	-1.18074	0.06619	0.34553	C	-0.541	-1.04503	0.517315
C	0.20059	0.28877	0.60776	C	0.256617	0.129289	0.622849
C	0.6346	1.59223	0.79903	C	-0.38089	1.346384	0.815062
C	-0.18622	2.71165	0.77626	C	-1.75393	1.510705	0.934303
C	-1.55936	2.50893	0.5593	C	-2.55575	0.358884	0.869909
C	-3.53719	0.95845	0.17827	C	-2.80297	-2.16056	0.644239
C	-4.02653	-0.44795	-0.0787	C	-2.13848	-3.4957	0.396495

C	-2.97687	-1.29683	-0.77408	C	-0.85233	-3.39166	-0.40378
C	-1.71725	-1.32116	0.08512	C	0.08379	-2.39805	0.276277
C	2.42969	0.35267	0.97151	C	1.752834	1.818169	0.727141
C	-2.43367	3.73811	0.55664	C	-4.04362	0.561039	1.016551
C	-3.49678	-2.71174	-1.01894	C	-1.10957	-3.04482	-1.87399
C	1.38067	-0.52687	0.74752	C	1.662633	0.441427	0.588265
C	1.48444	-2.00754	0.66789	C	2.808332	-0.49236	0.431007
C	3.89198	0.18334	1.17057	C	2.884331	2.777508	0.754125
O	4.53415	0.44746	-0.08431	O	3.690715	2.558498	-0.41772
C	4.79055	-0.63872	-0.85718	C	3.434725	3.138522	-1.61232
C	5.42691	-0.20949	-2.14383	O	4.14533	2.899358	-2.58491
O	4.54692	-1.80078	-0.56466	C	2.274468	4.084204	-1.70604
H	0.2347	3.69884	0.93417	H	-2.17474	2.499452	1.0825
H	-4.91808	-0.38251	-0.71399	H	-2.85021	-4.14183	-0.13094
H	-4.32533	-0.88302	0.88233	H	-1.94168	-3.94791	1.376104
H	-2.73838	-0.84542	-1.74721	H	-0.36484	-4.37553	-0.39356
H	-0.96401	-1.91452	-0.44286	H	0.403348	-2.79726	1.247335
H	-1.91012	-1.80657	1.04999	H	0.979142	-2.29645	-0.34455
H	-1.83851	4.65357	0.65177	H	-4.29828	1.62443	1.092007
H	-2.98131	3.8202	-0.38742	H	-4.57355	0.175714	0.139875
H	-3.12573	3.71913	1.40413	H	-4.40836	0.081948	1.930328
H	-2.7436	-3.31835	-1.53271	H	-0.1668	-3.00699	-2.43025
H	-4.39504	-2.69202	-1.64502	H	-1.74043	-3.8078	-2.34265
H	-3.7506	-3.21259	-0.07843	H	-1.60919	-2.07903	-1.99652
H	0.67146	-2.48506	1.22328	H	2.696004	-1.3594	1.089092
H	1.44203	-2.3368	-0.37486	H	2.877636	-0.83912	-0.60463
H	2.42047	-2.37162	1.10143	H	3.757696	-0.01303	0.690002
H	4.26225	0.9241	1.88751	H	2.560327	3.818924	0.833433
H	4.14886	-0.80222	1.57283	H	3.517671	2.572649	1.62361
H	6.37454	0.29481	-1.93882	H	2.433108	4.951444	-1.0616
H	5.6271	-1.09025	-2.76073	H	2.201171	4.45	-2.73574
H	4.74894	0.45103	-2.6902	H	1.337098	3.576099	-1.47507
**2b-2**	X axis(Å)	Y axis(Å)	Z axis(Å)	**2b-3**	X axis(Å)	Y axis(Å)	Z axis(Å)
O	0.32615	2.253947	-0.97527	O	0.513737	2.197776	0.851101
O	-3.90512	-2.18698	0.782583	O	-4.69721	-1.43671	0.291214
C	-1.91651	-0.98178	0.195503	C	-2.4624	-0.57086	0.261946
C	-0.488	-1.04848	0.158015	C	-1.08431	-0.92834	0.127738
C	0.225227	0.117667	-0.2407	C	-0.10593	0.076147	0.373364
C	-0.49919	1.239035	-0.61761	C	-0.5377	1.361065	0.667929
C	-1.88463	1.320569	-0.62722	C	-1.86774	1.746637	0.764271
C	-2.6065	0.190705	-0.20801	C	-2.8482	0.760874	0.562786
C	-2.68069	-2.18904	0.680957	C	-3.50449	-1.64687	0.082714
C	-1.91366	-3.41605	1.117116	C	-3.0654	-3.04343	-0.29506

C	-0.58627	-3.57833	0.398423	C	-1.74465	-3.08252	-1.04315
C	0.237408	-2.30657	0.571425	C	-0.68669	-2.33876	-0.23459
C	1.601791	1.813151	-0.81986	C	1.64786	1.467208	0.684362
C	-4.11053	0.309588	-0.21172	C	-4.28592	1.200907	0.685582
C	-0.76584	-3.96504	-1.07337	C	-1.87217	-2.55061	-2.47452
C	1.608553	0.501701	-0.36893	C	1.332909	0.143657	0.412165
C	2.816604	-0.31296	-0.07504	C	2.309274	-0.95687	0.201572
C	2.664549	2.793829	-1.15184	C	2.92246	2.213109	0.849266
O	3.493724	2.972399	0.010622	O	3.746394	1.88951	-0.28076
C	3.22623	3.872453	0.983586	C	4.981569	2.422403	-0.4213
O	3.969489	3.976766	1.955651	O	5.712697	2.082664	-1.34633
C	2.012234	4.739626	0.832538	C	5.417719	3.438648	0.593596
H	-2.37553	2.236395	-0.93858	H	-2.1219	2.775116	0.99709
H	-2.54083	-4.29713	0.936467	H	-3.85181	-3.49681	-0.91004
H	-1.75668	-3.3366	2.199575	H	-2.99321	-3.62325	0.633126
H	-0.03912	-4.40208	0.875684	H	-1.43093	-4.13167	-1.1246
H	0.524306	-2.19499	1.62492	H	-0.48586	-2.88087	0.698237
H	1.159685	-2.41926	-0.00592	H	0.24121	-2.3334	-0.81465
H	-4.43506	1.282746	-0.59773	H	-4.36164	2.281707	0.851347
H	-4.5555	-0.44852	-0.8637	H	-4.83461	0.988775	-0.23737
H	-4.50551	0.225334	0.805384	H	-4.76505	0.714127	1.540576
H	0.207804	-4.10884	-1.55401	H	-0.91166	-2.61855	-2.99649
H	-1.31914	-4.90661	-1.15733	H	-2.59917	-3.14355	-3.0399
H	-1.31098	-3.20645	-1.64328	H	-2.1976	-1.50639	-2.50721
H	2.755313	-0.74669	0.927846	H	2.009799	-1.85329	0.753107
H	2.91728	-1.12127	-0.80594	H	2.38287	-1.20278	-0.86222
H	3.732174	0.284405	-0.11824	H	3.308263	-0.68313	0.555374
H	3.303466	2.388127	-1.94329	H	2.730274	3.290555	0.873698
H	2.26859	3.744785	-1.51872	H	3.414193	1.893479	1.773668
H	1.103961	4.13644	0.800115	H	5.454969	2.995584	1.591098
H	1.936662	5.394254	1.707324	H	6.428543	3.777325	0.343705
H	2.104098	5.379732	-0.04741	H	4.761512	4.311392	0.567538

Table S2. The Cartesian coordinates of the lowest energy conformers for **3a** and **3b**

**3a-1**	X axis(Å)	Y axis(Å)	Z axis(Å)	**3a-2**	X axis(Å)	Y axis(Å)	Z axis(Å)
O	3.481569	-0.23996	0.475337	O	3.500168	-0.20538	0.506393
C	-0.61457	-0.1634	1.167837	C	-0.60509	-0.13033	1.132036
C	-0.10153	-0.03412	-0.15161	C	-0.07	0.00149	-0.1785
C	1.285674	-0.04382	-0.31697	C	1.320261	-0.01487	-0.32097
C	2.128803	-0.21886	0.768586	C	2.144253	-0.18532	0.777968
C	1.654022	-0.37255	2.055106	C	1.648516	-0.33737	2.056423
C	0.269155	-0.35507	2.268026	C	0.260478	-0.32265	2.246316
C	-2.11742	-0.17107	1.398034	C	-2.11183	-0.14049	1.33985

C	-2.92246	0.460123	0.263149	C	-2.90632	0.475984	0.189557
C	-2.47028	-0.10288	-1.05211	C	-2.41972	-0.07821	-1.11731
C	-1.01255	0.141295	-1.35101	C	-0.96374	0.200718	-1.38795
C	3.540472	-0.14815	-0.90702	C	3.596828	-0.09482	-0.87718
C	-0.24449	-0.52995	3.673149	C	-0.27558	-0.49577	3.643523
C	-3.28432	-0.78524	-1.87288	C	-3.20473	-0.77504	-1.95385
C	2.147344	0.113387	-1.51665	C	2.198964	0.116174	-1.5153
O	4.57447	-0.26287	-1.55607	O	4.661794	-0.16798	-1.47705
O	1.81705	-0.83414	-2.51976	O	1.880176	-0.86208	-2.49337
C	2.119686	1.520326	-2.10634	C	2.160923	1.499058	-2.15636
H	2.350026	-0.51246	2.876873	H	2.331276	-0.47263	2.890326
H	-2.3599	0.375744	2.316299	H	-2.36915	0.412276	2.250521
H	-2.43247	-1.21344	1.53624	H	-2.42441	-1.18313	1.482043
H	-2.77442	1.547736	0.254692	H	-2.77657	1.56597	0.183798
H	-3.99174	0.288605	0.43386	H	-3.97562	0.286815	0.340314
H	-0.68854	-0.52836	-2.1551	H	-0.61688	-0.43613	-2.20874
H	-0.9133	1.174106	-1.70533	H	-0.87563	1.246105	-1.70704
H	0.55842	-0.8163	4.361058	H	0.515855	-0.78388	4.343882
H	-0.67713	0.407547	4.036018	H	-0.71165	0.442906	3.999144
H	-0.99819	-1.3228	3.711736	H	-1.03159	-1.28688	3.670752
H	-2.92631	-1.19404	-2.81336	H	-2.82425	-1.17184	-2.89065
H	-4.32753	-0.95704	-1.62612	H	-4.24899	-0.96707	-1.72632
H	2.581346	-0.87377	-3.12472	H	1.72524	-1.70383	-2.0302
H	1.146856	1.751393	-2.55134	H	1.19239	1.701509	-2.624
H	2.346422	2.28616	-1.35605	H	2.367235	2.294819	-1.43179
H	2.855843	1.613887	-2.91358	H	2.907401	1.571877	-2.95627
**3b-1**	X axis(Å)	Y axis(Å)	Z axis(Å)	**3b-2**	X axis(Å)	Y axis(Å)	Z axis(Å)
O	0.472775	-3.48799	0.025875	O	0.495079	-3.50356	0.076995
C	1.197112	0.601917	-0.02724	C	1.153792	0.595198	-0.00874
C	-0.12493	0.099417	-0.17164	C	-0.1601	0.070951	-0.15092
C	-0.30713	-1.28399	-0.11511	C	-0.31905	-1.31616	-0.08919
C	0.771725	-2.13647	0.056365	C	0.771914	-2.14833	0.095636
C	2.059701	-1.67157	0.226048	C	2.051533	-1.66168	0.265384
C	2.283715	-0.2883	0.207266	C	2.25395	-0.27568	0.233967
C	1.443377	2.101899	-0.06214	C	1.377471	2.098657	-0.05207
C	0.34438	2.896676	-0.76612	C	0.269871	2.869151	-0.76862
C	-0.99958	2.473943	-0.24912	C	-1.0681	2.435829	-0.24582
C	-1.29938	1.012244	-0.46637	C	-1.35084	0.967403	-0.43528
C	-0.87972	-3.53645	-0.27846	C	-0.86076	-3.59063	-0.22291
C	3.691053	0.213586	0.397311	C	3.652668	0.249824	0.425574
C	-1.84288	3.316258	0.368389	C	-1.92257	3.27739	0.357281
C	-1.52069	-2.13344	-0.21917	C	-1.52046	-2.18641	-0.20761
O	-1.4842	-4.56836	-0.54868	O	-1.42362	-4.65092	-0.46364

O	-2.27261	-1.81795	-1.37879	O	-2.26009	-1.87996	-1.37767
C	-2.41766	-2.0699	1.015629	C	-2.44544	-2.12205	1.005495
H	2.876079	-2.37384	0.366135	H	2.87813	-2.3498	0.417148
H	2.38549	2.317386	-0.57885	H	2.318663	2.325328	-0.56562
H	1.543343	2.450856	0.973775	H	1.466331	2.456545	0.98178
H	0.382285	2.71527	-1.84806	H	0.310903	2.672954	-1.84784
H	0.51815	3.969255	-0.62009	H	0.429508	3.945811	-0.63726
H	-2.15417	0.723678	0.152182	H	-2.18873	0.6773	0.204852
H	-1.5817	0.873657	-1.51759	H	-1.65147	0.810569	-1.47874
H	4.35604	-0.5832	0.747814	H	4.330163	-0.53499	0.779124
H	4.087835	0.591046	-0.55021	H	4.045122	0.63135	-0.52212
H	3.719497	1.007276	1.150601	H	3.666318	1.045718	1.176951
H	-2.80701	2.982202	0.739905	H	-2.88232	2.937255	0.735245
H	-1.59312	4.362411	0.517097	H	-1.68731	4.329315	0.48853
H	-2.85614	-2.58204	-1.54448	H	-1.63209	-1.8416	-2.11997
H	-2.88276	-1.08678	1.132484	H	-2.93288	-1.14754	1.0991
H	-3.2405	-2.7896	0.929311	H	-3.25245	-2.85843	0.909982
H	-1.86836	-2.29909	1.935929	H	-1.91314	-2.32879	1.940983

2. NMR spectra of **1−3**, HRESIMS of **1** and **2**, and HREIMS of **3**

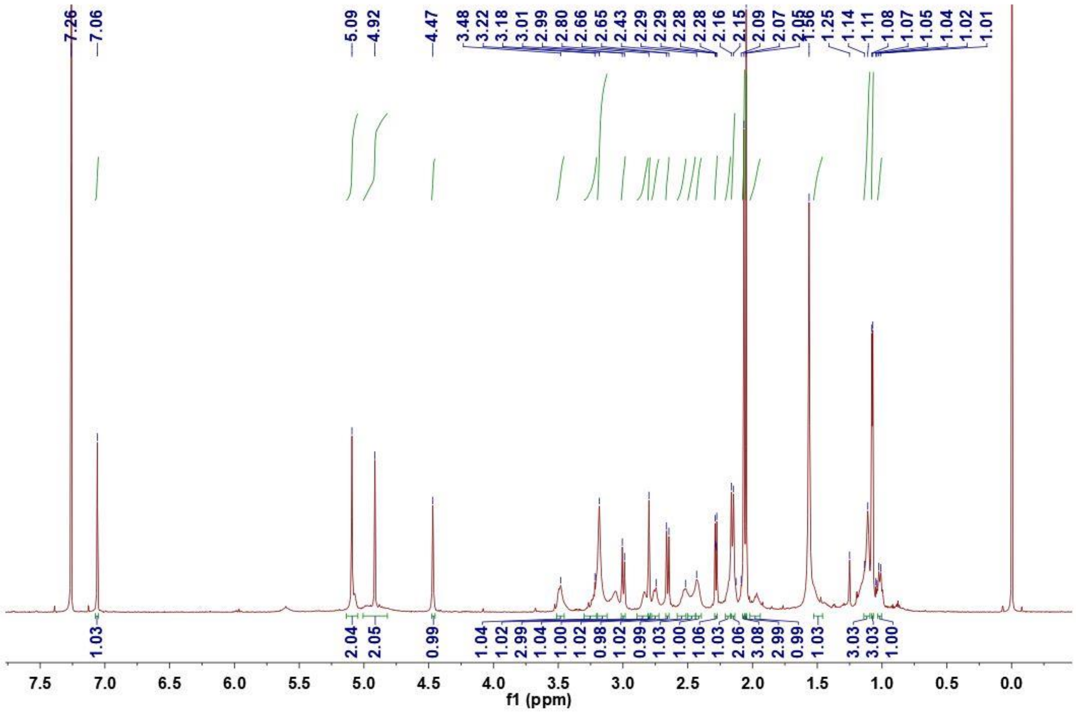

Figure S5.^{1}H NMR spectrum of **1** in $CDCl_3$.

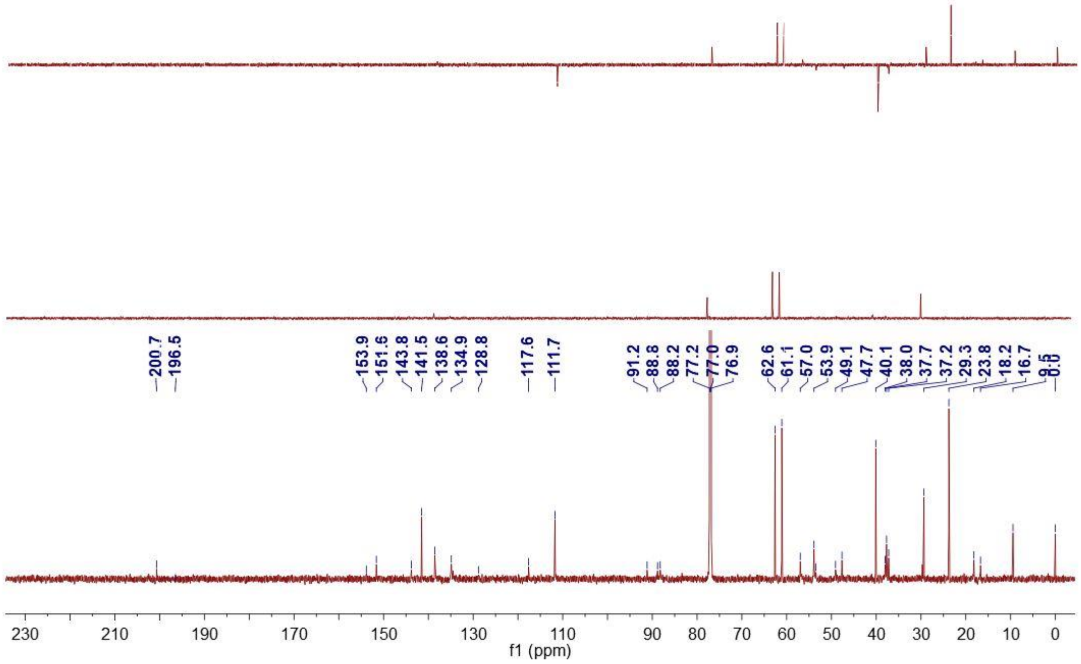

Figure S6. ^{13}C NMR and DEPT spectra of **1** in $CDCl_3$.

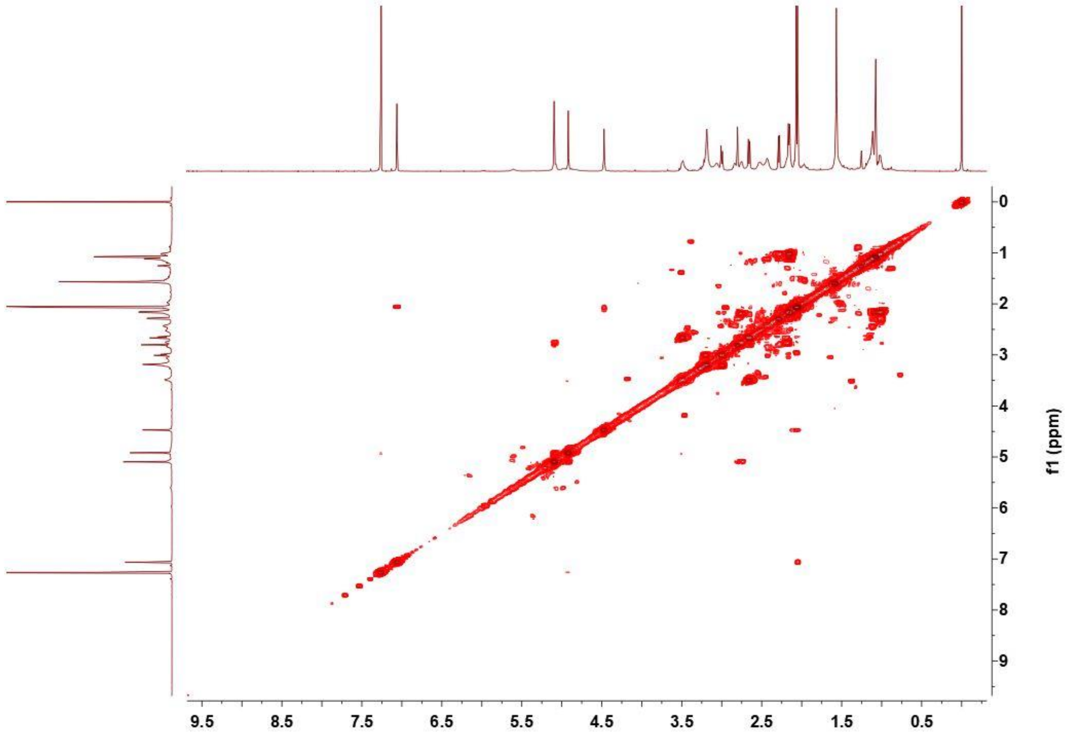

Figure S7. ^{1}H-^{1}H COSY spectrum of **1** in $CDCl_3$.

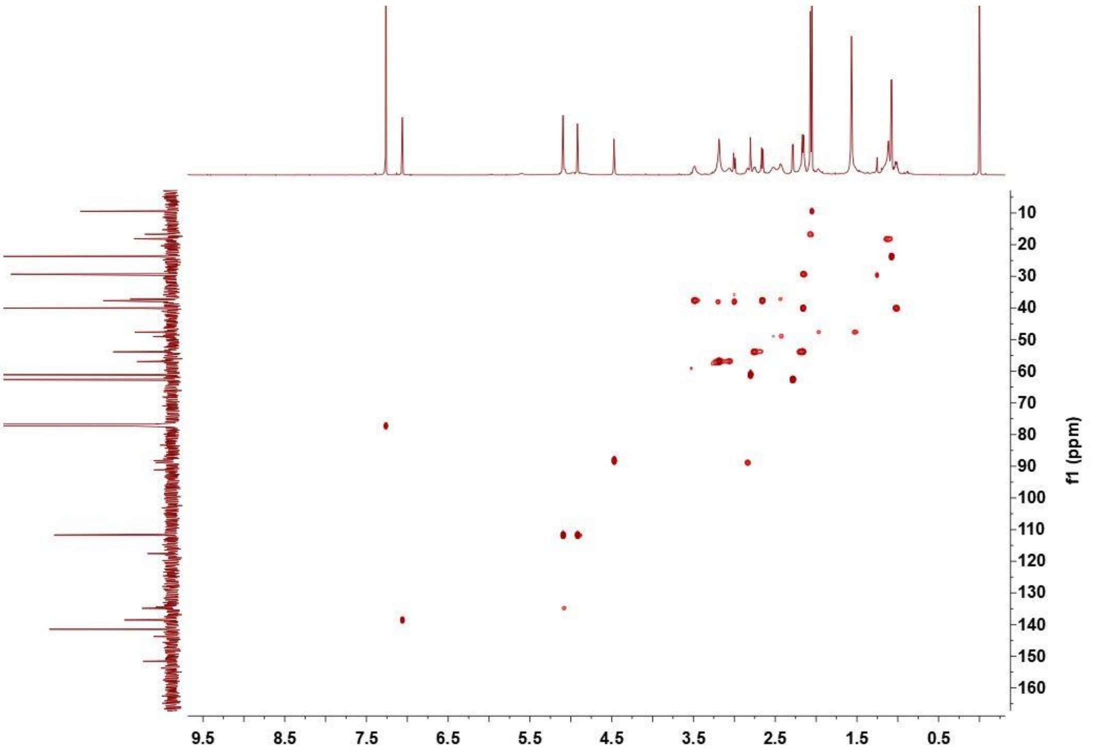

Figure S8. HSQC Spectrum of **1** in $CDCl_3$.

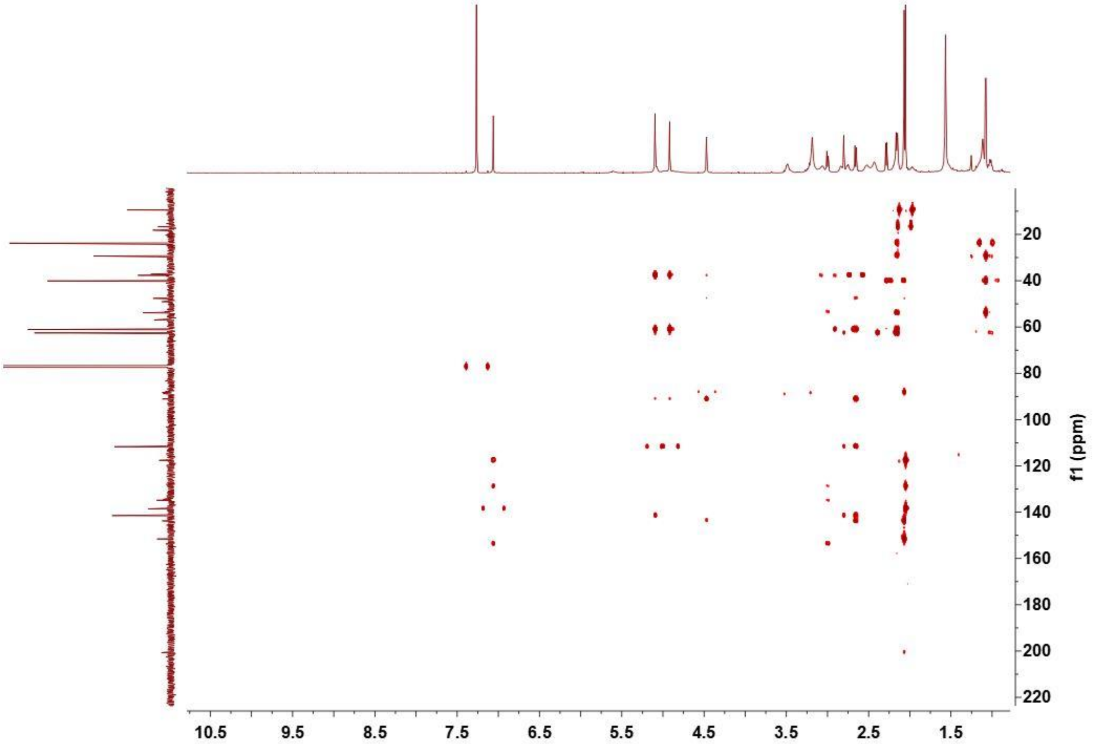

Figure S9. HMBC spectrum of **1** in $CDCl_3$.

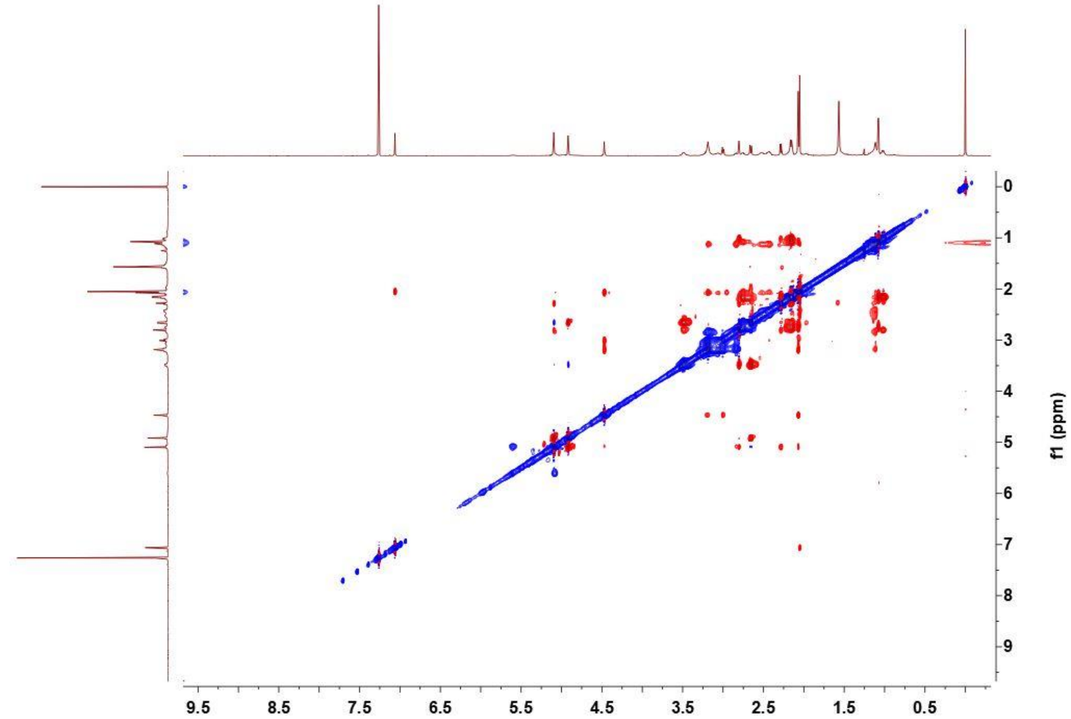

Figure S10. ROSY spectrum of **1** in $CDCl_3$.

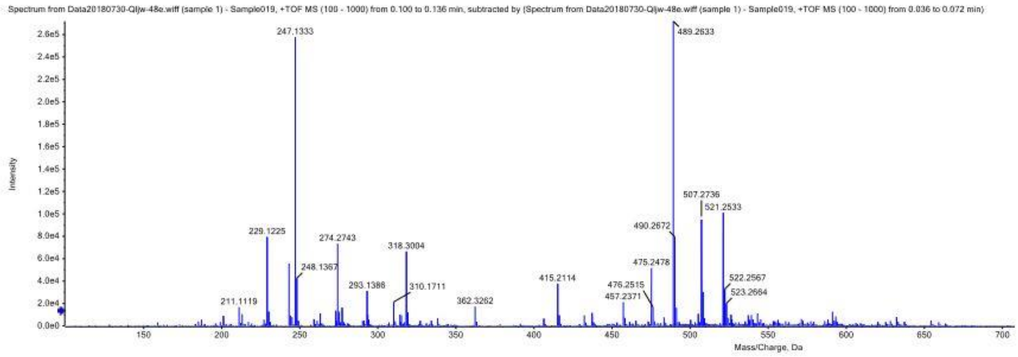

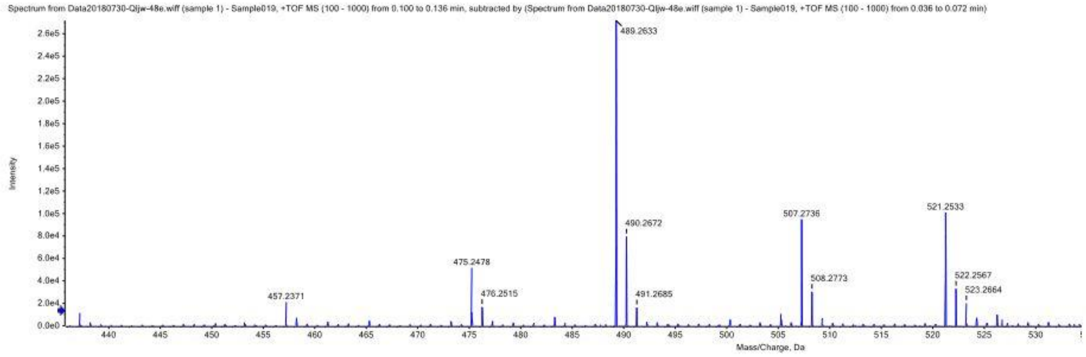

$[M+H]^+$ m/z 507.2736

Hit	Formula	m/z	RDB	ppm
1	$C_{31}H_{38}O_6$	507.2741	13.0	-1.0

Elments from ~ to C60H120O60

Figure S11. HRESIMS of **1**.

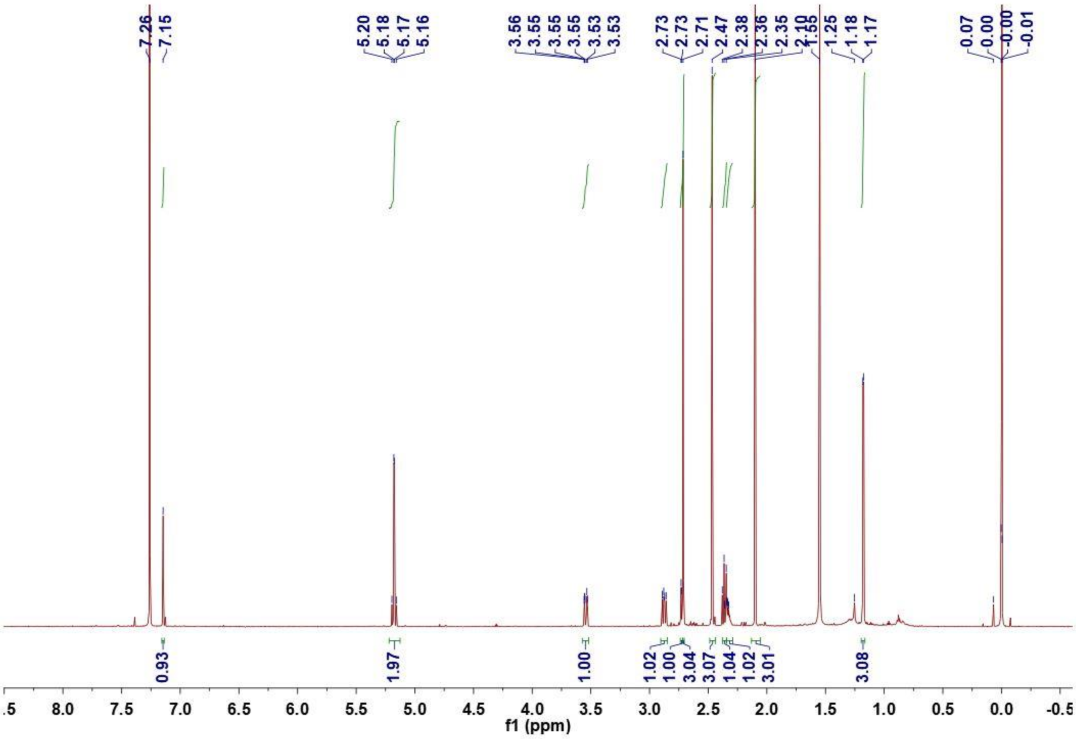

Figure S12.^{1}H NMR spectrum of **2** in $CDCl_3$.

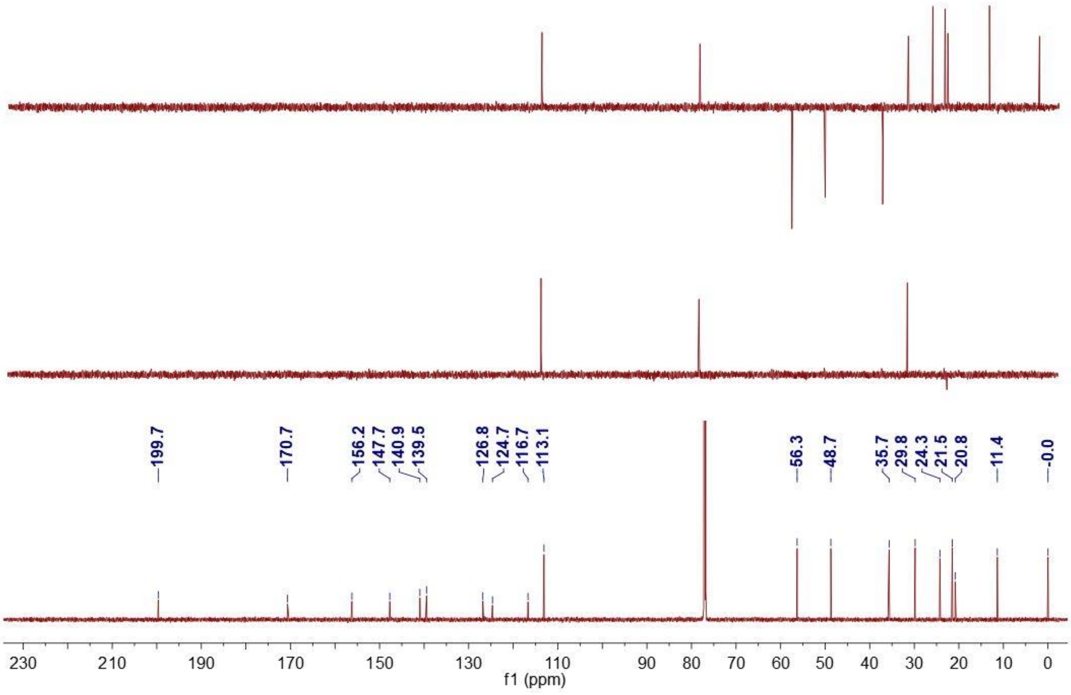

Figure S13. ^{13}C NMR and DEPT spectra of **2** in $CDCl_3$.

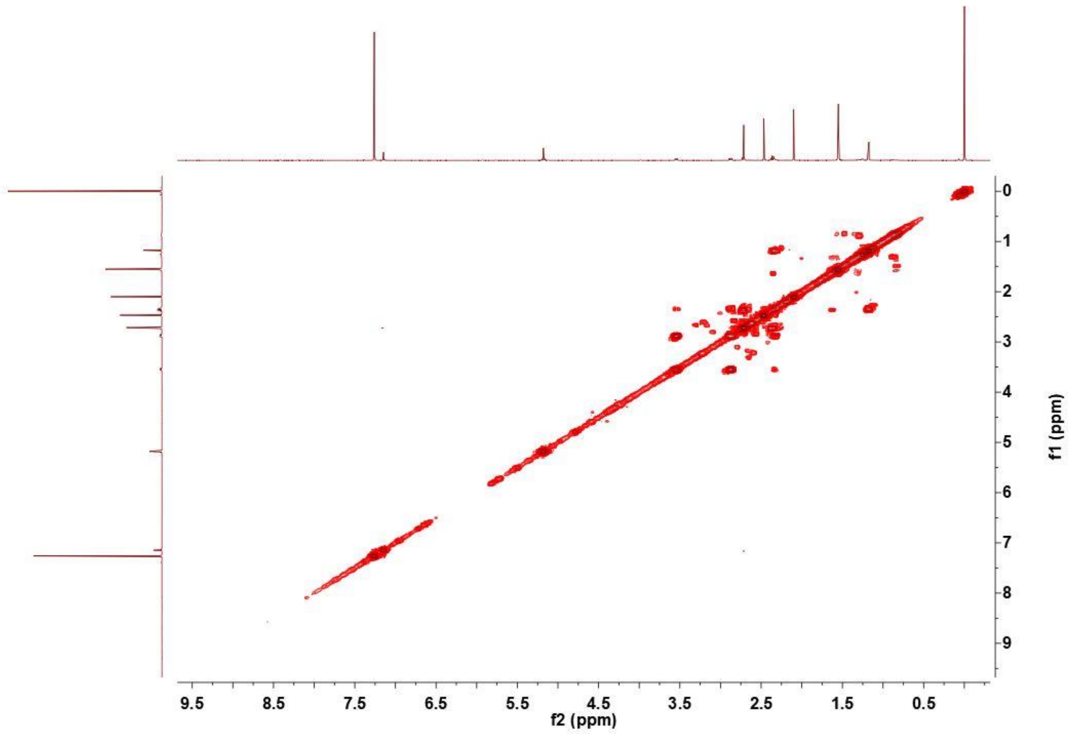

Figure S14. ^1H-^1H COSY spectrum of **2** in $CDCl_3$.

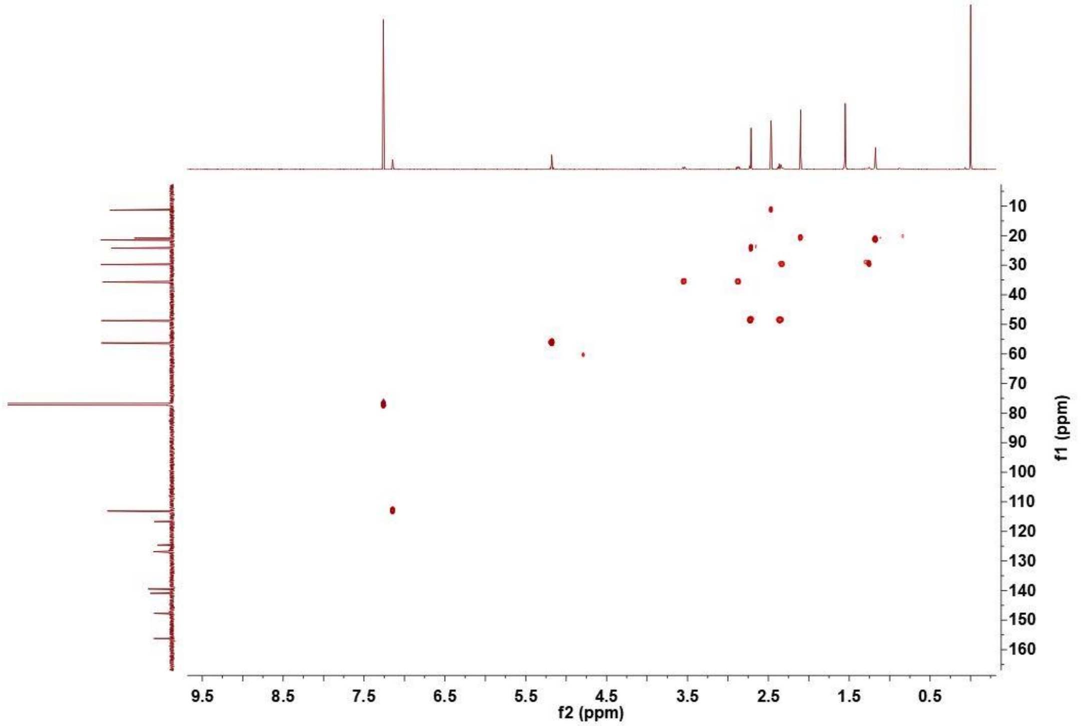

Figure S15. HSQC spectrum of **2** in $CDCl_3$.

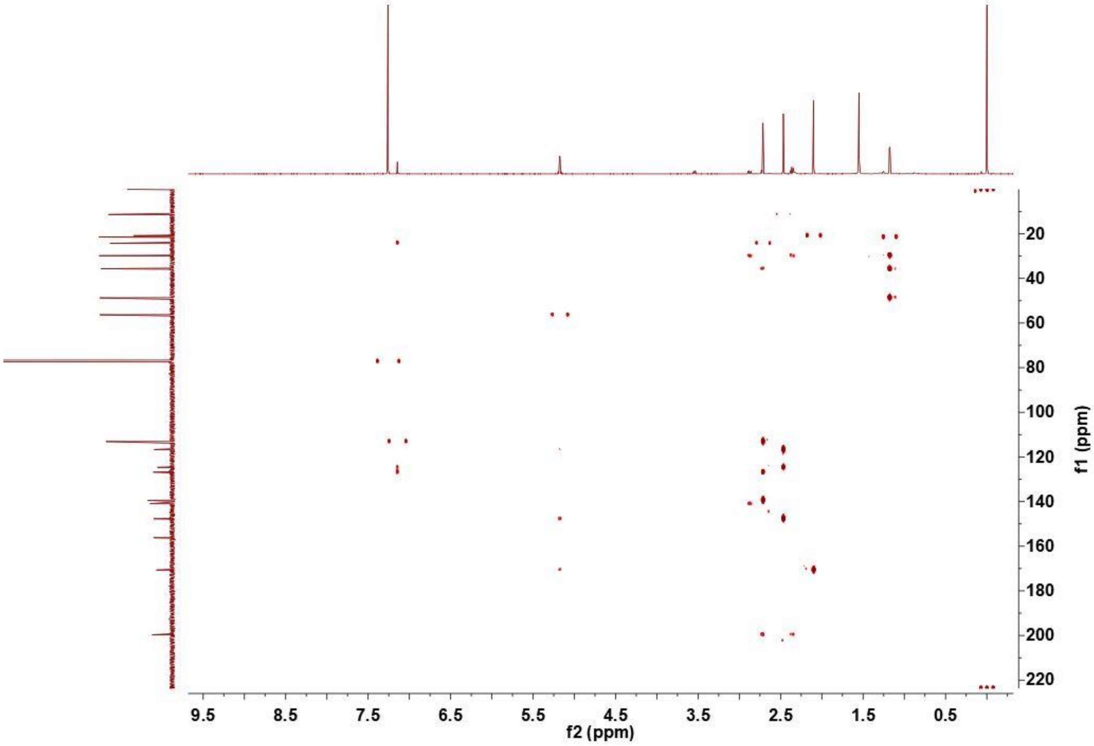

Figure S16. HMBC spectrum of **2** in $CDCl_3$.

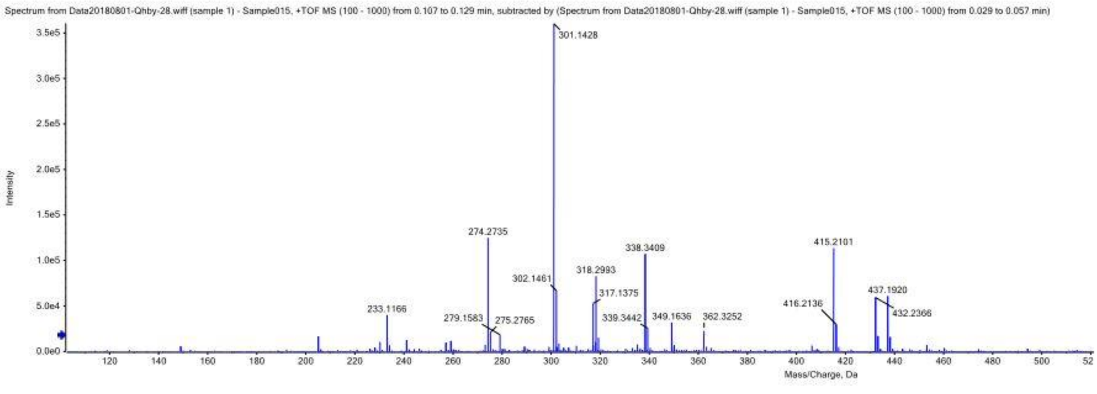

$[M+H]^+$ m/z 301.1428

Hit	Formula	m/z	RDB	ppm
1	C18H20O4	301.1434	9.0	-2.1

Elments from ~ to C60H120O60

Mass tolerance 5 ppm

Figure S17. HRESIMS of **2**.

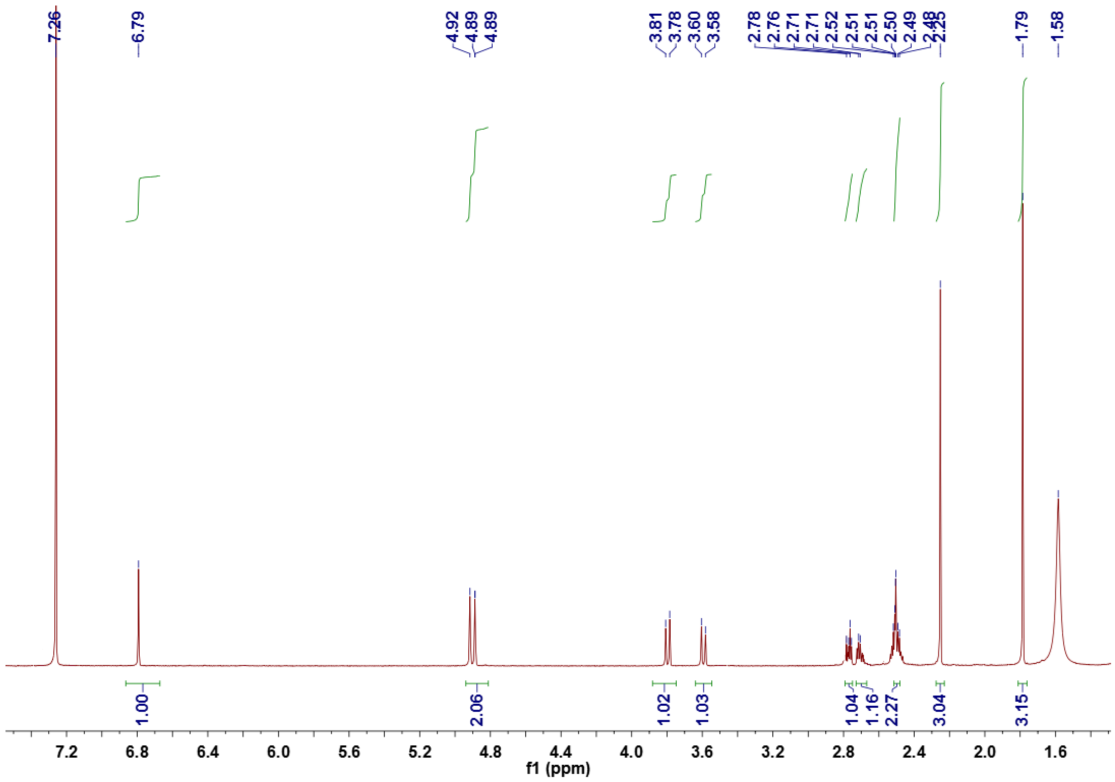

Figure S18. ^{1}H NMR spectrum of **3** in $CDCl_3$

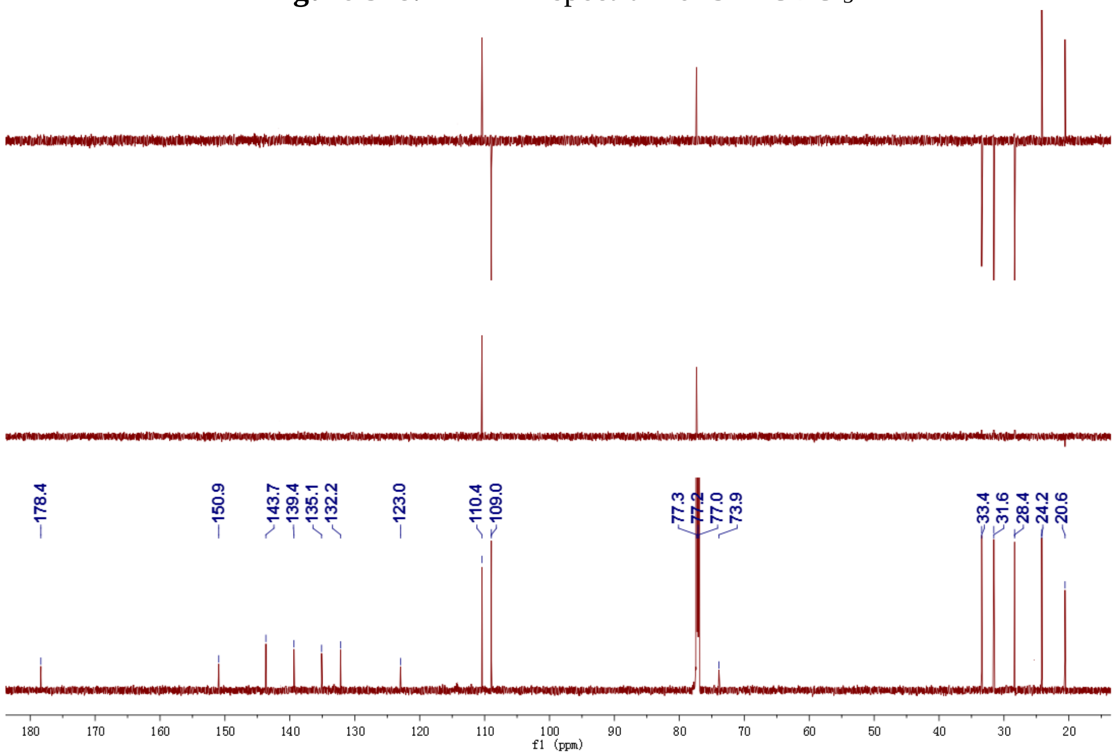

Figure S19. ^{13}C NMR and DEPT spectra of **3** in $CDCl_3$

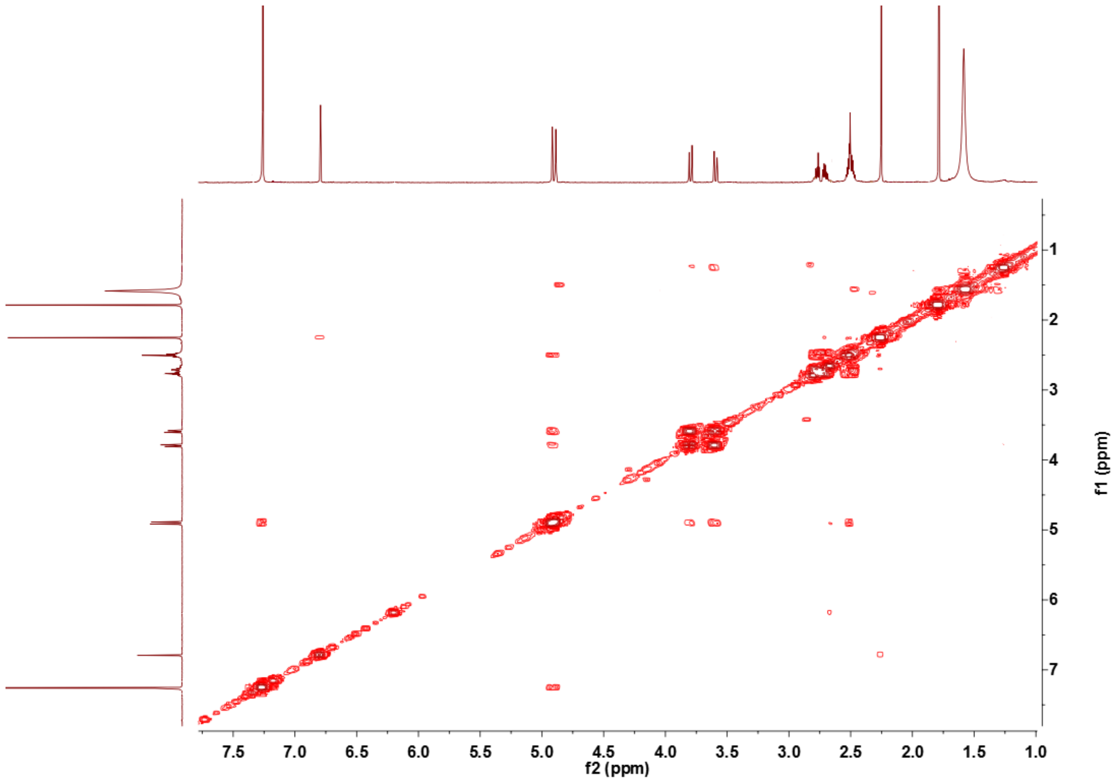

Figure S20. ^{1}H-^{1}H COSY spectrum of **3** in $CDCl_3$

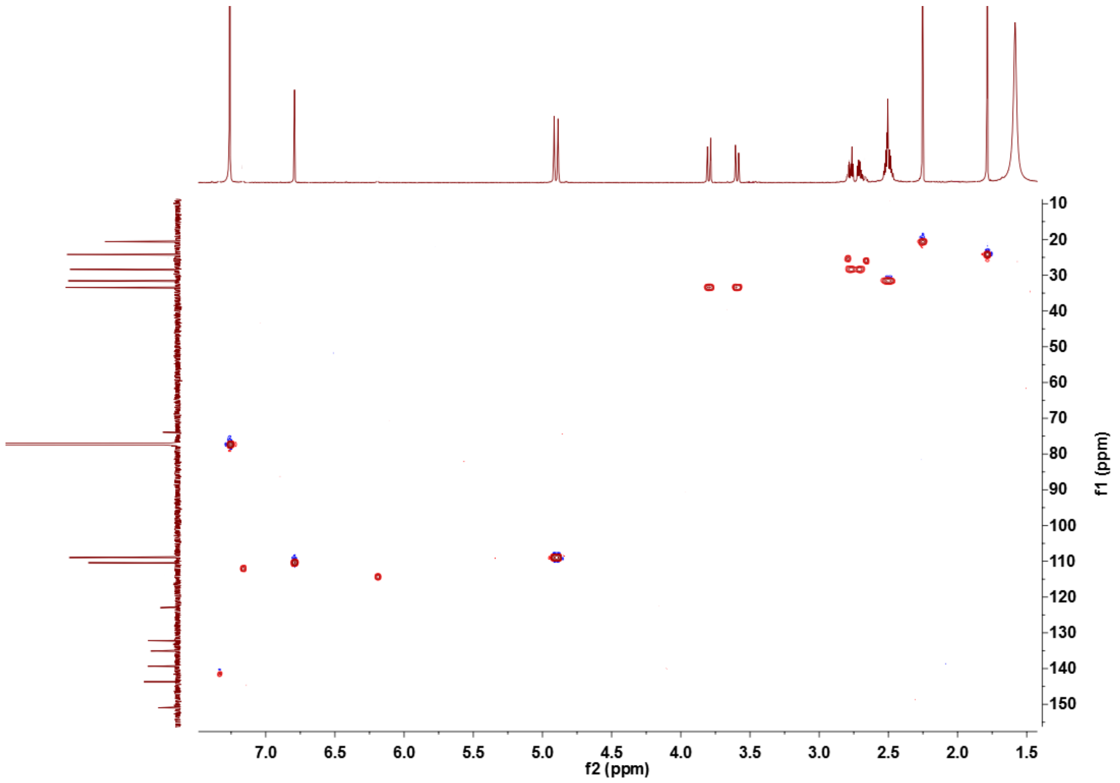

Figure S21. HSQC spectrum of **3** in $CDCl_3$

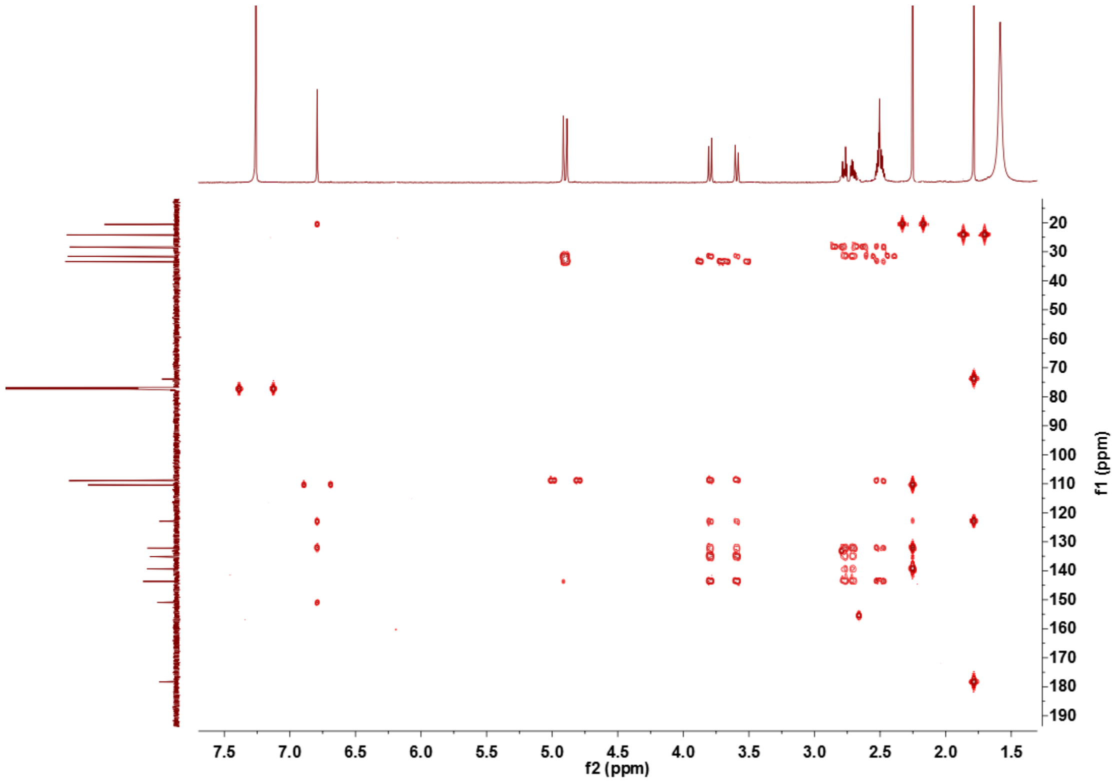

Figure S22. HMBC spectrum of **3** in $CDCl_3$

Elemental Composition Report **Page 1**

Single Mass Analysis
Tolerance = 10.0 PPM / DBE: min = -10.0, max = 120.0
Selected filters: None

Monoisotopic Mass, Odd and Even Electron Ions
14 formula(e) evaluated with 1 results within limits (up to 51 closest results for each mass)
Elements Used:
C: 0-200 H: 0-400 O: 2-4

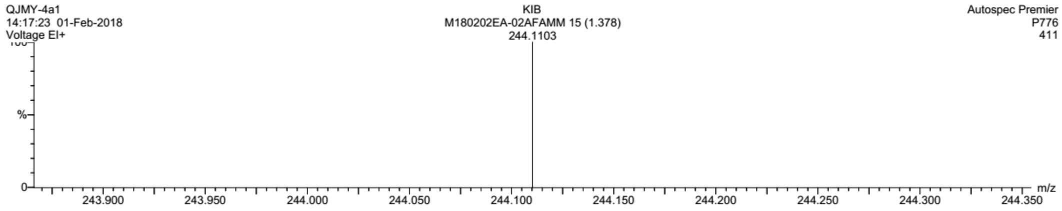

Minimum:				-10.0		
Maximum:		200.0	10.0	120.0		
Mass	Calc. Mass	mDa	PPM	DBE	i-FIT	Formula
244.1103	244.1099	0.4	1.6	8.0	5546209.5	C15 H16 O3

Figure S23. HREIMS of **3**
